# Anthropogenic marine litter composition in coastal areas may be a predictor of potentially invasive rafting fauna

**DOI:** 10.1371/journal.pone.0191859

**Published:** 2018-01-31

**Authors:** Sabine Rech, Yaisel J. Borrell Pichs, Eva García-Vazquez

**Affiliations:** Department of Functional Biology, University of Oviedo, Oviedo, Asturias, Spain; Auburn University, UNITED STATES

## Abstract

Anthropogenic plastic pollution is a global problem. In the marine environment, one of its less studied effects is the transport of attached biota, which might lead to introductions of non-native species in new areas or aid in habitat expansions of invasive species. The goal of the present work was to assess if the material composition of beached anthropogenic litter is indicative of the rafting fauna in a coastal area and could thus be used as a simple and cost-efficient tool for risk assessment in the future. Beached anthropogenic litter and attached biota along the 200 km coastline of Asturias, central Bay of Biscay, Spain, were analysed. The macrobiotic community attached to fouled litter items was identified using genetic barcoding combined with visual taxonomic analysis, and compared between hard plastics, foams, other plastics and non-plastic items. On the other hand, the material composition of beached litter was analysed in a standardized area on each beach. From these two datasets, the expected frequency of several rafting taxa was calculated for the coastal area and compared to the actually observed frequencies. The results showed that plastics were the most abundant type of beached litter. Litter accumulation was likely driven by coastal sources (industry, ports) and river/sewage inputs and transported by near-shore currents. Rafting vectors were almost exclusively made up of plastics and could mainly be attributed to fishing activity and leisure/ household. We identified a variety of rafting biota, including species of goose barnacles, acorn barnacles, bivalves, gastropods, polychaetes and bryozoan, and hydrozoan colonies attached to stranded litter. Several of these species were non-native and invasive, such as the giant Pacific oyster (*Crassostrea gigas*) and the Australian barnacle (*Austrominius modestus)*. The composition of attached fauna varied strongly between litter items of different materials. Plastics, except for foam, had a much more diverse attached community than non-plastic materials. The predicted frequency of several taxa attached to beached litter significantly correlated with the actually observed frequencies. Therefore we suggest that the composition of stranded litter on a beach or an area could allow for predictions about the corresponding attached biotic community, including invasive species.

## Introduction

Since plastics have been made available to a broad spectrum of consumers after the Second World War, their global production has risen to 322×10^9^ kg in 2015 [1]. Although plastic production is concentrated in China, Europe, the USA, Canada and Mexico, plastics and recyclable plastic waste, which are not classified as hazardous [2], are exported internationally [1,3,4], posing a global threat to human health, interests, and ecosystems [2,5]. The pollution by plastic litter has advanced to such a level that today it is present in virtually every environment and every location of the Earth [6,7]. The marine environment is especially affected, as it receives not only direct pollution from sea-based activities, but also land-based plastics [7–9]. Plastic pollution causes the death of a high number of marine animals, as well as severe damages to ecosystems and human health and interests, like tourism, fishing, or leisure activities at beaches [10–13]. Plastics do not degrade naturally but fragment to smaller pieces, which multiplies their abundance [6]. In recent decades, campaigns are being conducted to combat the excessive production and consumption of single-use plastics, for example plastic bags from supermarkets, microbeads in cosmetic products, or PET (Polyethylene terephthalate) beverage bottles (e.g. http://storyofstuff.org/, http://www.beatthemicrobead.org/). Policy changes have been requested after increasing scientific evidence and public awareness about the pollution problem [14,15].

While research and actions on several aspects of the plastic litter problem are steadily advancing, there are still many important aspects that have gained little scientific attention so far. One problem that has received less attention is the role of anthropogenic litter items serving as artificial rafts for non-native and possibly invasive species. Notably, rafting has been mentioned in several publications [16] and public media, but at present there is no clear understanding of the scale and the underlying processes of this phenomenon. Research priorities include an estimation of its global impact, the localization of natural sink areas, and the identification of high-risk anthropogenic litter items/materials and sources [17].

Rafting of biota on floating objects, like driftwood, macro algae or volcanic pumice has importantly shaped the species composition of islands [16,18,19]. Floatable litter items of anthropogenic origin greatly enhance the number of stable rafts, particularly in areas where natural vectors are scarce. Anthropogenic litter pollution is estimated to double marine rafting opportunities [16,20] and on some beaches more than 60% of all anthropogenic litter items carried attached organisms [6]. Although the vast majority of anthropogenic litter used as rafts are plastic items, there are also cases of macrobiotic rafting on glass, metal, and paper objects [16]. Notably, a metal gas cylinder encrusted by the stony coral *Favia fragum* had probably crossed the Atlantic Ocean from the USA to the Netherlands [21]. Another invading coral, *Oculina patagonica*, is commonly found on submerged metal objects [22], while some pelagic barnacles are frequently recorded on glass and metal objects [23]. Biofouling was also reported for air-filled glass floats, used in (mainly Japanese) fisheries before plastics became widely available and still afloat in the world´s oceans nowadays [21,24–26].

Differences between materials in the abundance and composition of the micro fauna in early stages of biofouling have been found [27,28]. Particularly, polystyrene seems to carry a higher number of both species and individuals than other types of plastics, which may be due to its higher surface roughness [27,29]. Settlement of individuals of the invasive species *Bugula neritina* was significantly higher on several plastic surfaces [Polyvinylchloride (PVC), Polypropylene (PP), Polycarbonate (PC), Polyethylene terephthalate (PET) and Polystyrene (PS)] than on glass surfaces, under both field and laboratory conditions, whereas the invasive barnacle *Austrominius modestus* settled more on glass than on plastic surfaces (tested under field conditions) [30]. In contrast, no significant differences between biofilm composition on PET and glass surfaces were found in another study and object softness, rather than the type of material, was suggested to be an important factor for biota attachment [31]. On the other hand, laboratory experiments and controlled field studies with fixed floaters do not incorporate the buoyancy or floating behaviour of the different materials, which may also influence the biotic colonization by some taxonomic groups [16,27,29,32]. The ability of items to float over long distances depends not only on their buoyancy, but also on their stability and shape, with thinner and more flexible plastic items (like plastic bags and packaging material) sinking faster than thicker and more robust plastic items [33].

The origin of litter could have an influence in the attached biota. Marine anthropogenic litter stems from various sources, like households, beach-based leisure activities, sea-going activities, industries, and sewage [34]. The contribution of each source to anthropogenic litter has been investigated at many locations [9,35–37], but the main sources of litter rafts with biota are less known. For particular items, macroscopic attached biota has been reported. Examples are lines, ropes, nets and bait pots [38–40], aquaculture and other buoys [39,41], plastic packaging bands used in Antarctic bases and fishing boats [42], virgin plastic pellets [43], glass bottles [39], a gas cylinder reported above [21], a plastic spool [40], and tennis shoes and slippers [44], amongst others. Those reports might point to a higher contribution of litter items originated from sea-based activities such as aquaculture and fisheries. However, this first impression needs to be investigated in depth and on a larger geographic scale.

Floating objects displace along with currents and tides, thus their role in the dispersal of attached species may be important. Rafting on marine litter has been suggested to be involved in regional dispersal of several invertebrates [23,45,46]. For example, juveniles of the bivalve *Pinctada imbricata* and adults of *Isognomon bicolor*, which are considered invasive in Brazil, were found attached to anthropogenic litter for the first time at the Uruguayan coast, where they are regarded as potentially invasive as well [38,44]. In the Spanish part of the Bay of Biscay, several alien invasive species are registered [47], some of which are already known to attach to floating anthropogenic litter in other regions [16]. The invasive pygmy mussel *Xenostrobus securis* was first reported in the Bay of Biscay in 2012, attached to natural as well as plastic and metal objects, among others [48]. The invasive *Crassostrea gigas* and the exotic *Ostrea stentina* were also found attached to artificial materials on regional ports [49]. According to EU Regulation (EU) No 1143/2014 there are about 12,000 alien species in European countries, of which 10–15% are regarded as invasive and pose a serious threat to the environment and human interests [50]. Such species can be regarded as ecosystem infestations or epidemics, with the anthropogenic litter carrying it, being infested vectors.

Given the concern of anthropogenic beach litter our goal was to determine whether the composition of anthropogenic beach litter can predict macrobiotic communities attached to stranded litter items in a region. In answering this goal, we had three main objectives. First, determine which native, non-native, and potentially invasive macroscopic animal species are present on stranded anthropogenic litter items. Second, determine the principal material and sources of the infested vectors. Third, test if the occurrence of a certain species/ taxon can be predicted based on the general litter composition at a beach or a coastal area.

## Material and methods

No specific permissions were required for sampling because all the organisms analysed in this study were obtained from litter items. Those items must be removed from the beaches as they are not natural substrate. The field studies did not involve endangered or protected species.

### Sampling area

**Fig 1:**
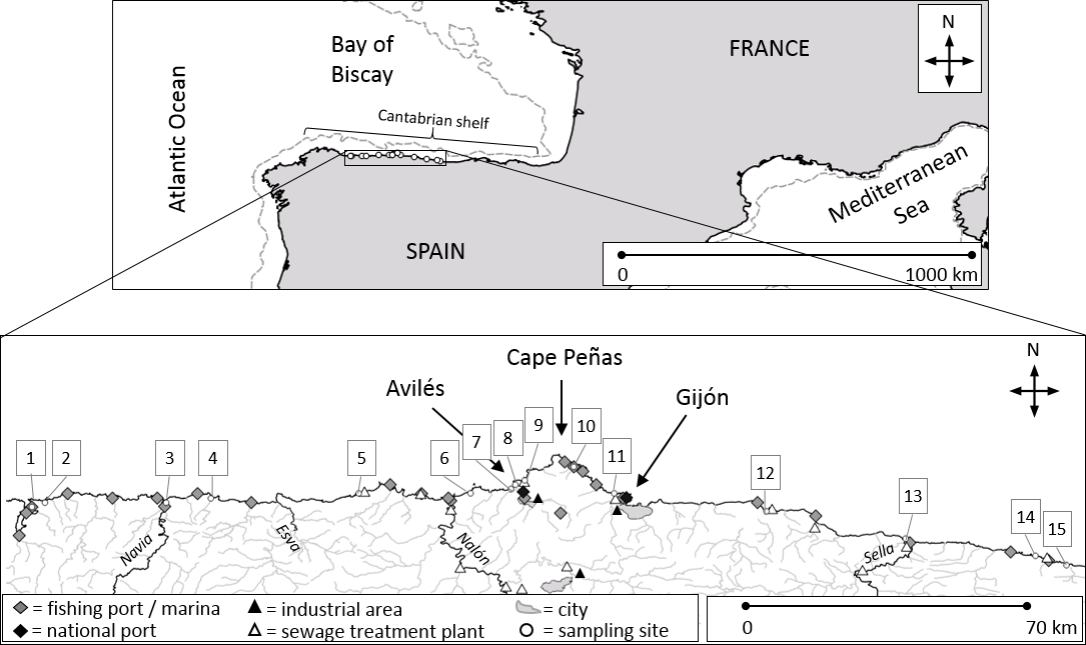
Map of the sampled area including waterways, national ports, fishing ports and marinas, sewage treatment plants, and principal industrial sites. Sampling sites are numbered and are specified in Table 1.

To address our main research goal and objectives, we evaluated the coast of Asturias region in the south-central Bay of Biscay (north of Spain). The coast is under the influence of currents going eastwards [51], with a boundary in Cape Peñas (central cape marked in Fig 1) that divides the coast into the colder west and the warmer east zone [52]. The sampling sites cover a wide spectrum of factors that may influence marine litter distribution, like land-use, distance to human settlements, industry, and geomorphology [53–55]. There are two international cargo ports in the sampled area (Gijón and Avilés), as well as shellfish aquaculture areas in two estuaries (Ría del Eo and Villaviciosa). There are several villages and two bigger cities, Gijón and Avilés, along the coastline in Spain (Fig 1). The central area of the region is strongly polluted by industrial activities [56,57], which are mainly based in the area of Avilés. Among the several rivers discharging into the Cantabrian Sea in the sampling area, the rivers Nalón, Navia, Sella, and Esva have the largest stream basins (Fig 1).

**Table 1:** Sampled beaches as shown in Fig 1, with geographic position.

Number	Beach name	Longitude [°W]	Latitude [°N]
1	Figueras	-7.02	43.54
2	Penarronda	-6.99	43.55
3	Navia	-6.72	43.55
4	Barayo	-6.62	43.56
5	Silencio	-6.29	43.57
6	Bayas	-6.04	43.57
7	Salinas	-5.95	43.58
8	Nieva	-5.94	43.59
9	Xagó	-5.92	43.60
10	Bañugues	-5.81	43.63
11	Xivares	-5.72	43.57
12	Rodiles	-5.38	43.53
13	Sta. Marina	-5.07	43.47
14	Poo	-4.78	43.43
15	Andrín	-4.71	43.41

### Beach litter samplings and analysis

A total of fifteen sandy beaches, covering a linear distance of 190 km along the Cantabrian coastline in Asturias, Spain, were sampled in a 26-day period between February and March 2016 (Fig 1). Each beach was sampled one day during low tide and daylight. We conducted two independent surveys: 1) A sampling of fouled beached items along the whole area of each beach to test if there are material-related differences in the taxonomic composition of the macro fauna attached to beached litter, and 2) a count and material-based classification of beached anthropogenic litter in general (both fouled and non-fouled) in a smaller standardized area. Please see the supporting figure for a graphic sampling scheme (S1 Fig).

Survey 1: The whole area of each beach was searched for anthropogenic litter items with attached macrofauna (visible fauna). Each of the items found was photographed with a Motorola Moto G3 camera (resolution 13 MP) next to a size reference (a finger or any other object of known dimensions) and given an identification code. The type of object (e.g. buoy, fragment, rope; Table 2), type of material and colour was noted down for each item. We did not only classify the fouled items by material as plastic and non-plastic (e.g. metal, paper, glass; abbreviated NPl), but moreover separated plastic items in three categories, based on their stability and surface roughness: Hard plastics (abbreviated HPl), synthetic foams (e.g. Polystyrene; abbreviated foams), and other plastics (abbreviated OPl). Litter items found on the beaches were associated to three sources: Sewage, Fishing/Aquaculture and Household/Leisure. All objects or fragments that were not identifiable or not attributable to one of the categories above were classified as N/A (not attributable; Table 2).

**Table 2:** Categories of beach litter sources and associated litter objects.

Sewage	Fishing/Aquaculture	HH/Leisure	N/A
Cotton buds	Buoys	Sandals	Fragments
Menstrual hygiene	Netfloats	Cosmetics container	Unknown objects
products + packaging	Cage nets	Shoes	Boxes
Wet wipes	Jerrycans	Shoe soles	Bottles
	Nets	Cigarette stubs	Buckets
	Ropes	Lighters	Lids
		Paper and carton	Beverage crates
		Textiles	
		Drinking straws	

HH = household, N/A = not attributable.

Attached biota was visually assigned to the most specific distinguishable taxonomic group based on morphology and the number of individuals (colonies for bryozoans and hydrozoans) was counted and noted down for each group. A representative number of individuals (≤ 50) of each morphotype was detached from each litter item using forceps and a scraper. They were stored in commercially available hard plastic sampling pots in 50–500 ml (depending on the size and number of stored individuals) of ethanol 80% for further analysis and labelled with the identification code of the corresponding litter item. Some smaller litter items and items of complex shapes were stored in plastic bags and taken to the laboratory for measurement, while the dimensions of bigger items and of items with a simple shape were estimated based on the photos, and the surface area was calculated for each item. The native distribution area and the potential invasive capacity of each attached species were examined from relevant current literature [49,58–62] and databases, namely the global invasive species database (GISD, http://www.issg.org/database) and World Register of Marine Species [63].

Survey 2: A standardized quantification and characterization of anthropogenic beach litter (not restricted to fouled objects) was done at all beaches, except for Figueras, Silencio, and S. Juan de Nieva (for location of the beaches see Fig 1). On the other 12 beaches, of similar sandy granulation, standardized litter counts were conducted in 2 horizontal transects at every beach, each consisting of four adjoined quadrats of 3×3m^2^ each. The two transects were placed parallel to the water line, the upper transect along the most recognizable higher tideline, and the lower transect along the most recognizable lower tideline, to account for possible differences in litter composition with shore height [64] and to include both recently stranded litter (lower tide line) and litter stranded less recently (most recognizable high tide line). The area for the counts was defined at every beach after visual inspection, where accumulation of flotsam (both natural and anthropogenic) was representative of the whole beach (i.e. neither exceptionally high, nor exceptionally low, relating to the rest of the beach). This method was chosen over a random approach to avoid bias due to the small transect area (36 m^2^ per transect) and the limited number of replicates (two transects per beach), as anthropogenic litter and other flotsam is often distributed heterogeneously along the beach [64,65].

The sampling quadrats were defined with a tape measure and their outlines were marked in the sand using a stick. In each quadrat all macro litter (items and fragments bigger than 1.5 cm) was inspected and sorted by object type (e.g. lid, drinking straw, fragment) and material. Then the number of items of each combination of object type and material (e.g. hard plastic lids, metal lids, paper fragments; Table 2) was counted and noted down for each quadrat in situ. All items and fragments were then assigned to a source category. The material categories and source categories used for classification were the same as described above for Survey 1.

### Genetic barcoding

DNA was extracted from a small piece of tissue (about 2×2 mm) using Chelex (Bio Rad BT Chelex® 100 Resin). For DNA extraction from very small individuals with non-tissue parts, like shells (e.g., molluscs), the complete individual was treated with E.Z.N.A® Mollusc DNA Kit. PCRs were performed with the universal primers detailed in Table 3. When necessary, the PCR product was purified using EUR_x_® Gene Matrix Agarose Out DNA Purification Kit. DNA sequencing was performed by Macrogen Europe, Amsterdam, Netherlands.

**Table 3:** Primers used for DNA amplification in different taxa.

Taxon	Primers	Sequence
Molluscs, Arthropods	jgLCO1490 jgHCO2198	^5’^TITCIACIAAYCAYAARGAYATTGG^3’^ ^5'^TAIACYTCIGGRTGICCRAARAAYCA^3'^
Polychaetes	18s EukF 18s EukR	^5’^WAYCTGGTTGATCCTGCCAGT^3’^ ^5’^TGATCCTTCYGCAGGTTCACCTAC^3’^
Bryozoans Hydrozoans	16s HF 16s HR	^5’^ATAACACGAGAAGACCCT^3’^ ^5’^CCCRCGGTCGCCCCAAC^3’^

Sequence editing and alignment was done using the freeware BIOEDIT Version 7.2.5 [66]. From the DNA Barcode the species was assigned using the BLAST database [67] and the best match with the maximum hit score (minimum 97% nucleotide identity). Phylogenetic trees for confirming species assignation were built with MEGA 7 [68] from the sequences obtained in this study and reference sequences of voucher specimens taken from GenBank (https://www.ncbi.nlm.nih.gov/nucleotide/), based on the maximum likelihood reconstruction method, with 500 bootstraps.

### Statistical analysis

Analysis of rafting fauna was done at regional level after confirming large dispersal capacity of the species found. Comparison among materials for the attached biotic community was done using the number of individuals per object as a standardized unit. To compare among communities we classified biota as goose barnacles, acorn barnacles, bryozoan and hydrozoan colonies, decapods, molluscs and polychaetes.

Composition and sources of beach litter found along the main accumulation lines (from standardized samplings) were compared to composition and sources of the litter items used as rafts, employing the PERMANOVA function of PRIMER 6 software [69,70]. PERMANOVA results were regarded as statistically significant at a *p*-value of ≤ 0.05. The contribution of each litter source to the differences was tested by SIMPER (= similiarity percentage) analysis. Both analyses were based on Bray- Curtis similarities.

The abundance of anthropogenic litter was compared between and within beaches using boxplots, showing the mean value, quartiles and variability for each beach. Heterogeneity in composition and abundance of anthropogenic beach litter in general, and of items used as artificial rafts by biota, were tested using PERMANOVA, based on Euclidean distances. Multidimensional scaling (MDS) based on Bray-Curtis similarities was used to graphically represent the grouping of the sampled beaches, based on dominant litter material: beaches dominated by hard plastics (termed HPl–dominant), beaches dominated by other plastics (termed OPl-dominant), and beaches with mixed litter composition and less than 25 litter items in the standardized sampling area (> 0.35 items×m^2^; termed Mix). These analyses were done for the subsample of beaches where standardized litter analysis was carried out.

Since litter composition and litter with rafting biota in a beach were independent datasets, a correlation approach was followed to determine if rafting biota in a beach area can be inferred from litter composition. Biota expectation from litter composition was estimated for 12 beaches based on the characteristic community profile of the beaches’ litter materials. The goodness of adjustment between estimated and observed taxa was tested using a correlation approach, based on Spearman´s rank correlation coefficient and the linear correlation was graphically illustrated in a scatter plot.

We calculated the expected number of individuals by taxa at each of the twelve beaches as:

$$TB(x)=\sum_{i=1}^{n}fM(i)*fTBM(i,r)*Nt(x)$$ (1)

Where *TB* (x) is the expected number of individuals for taxon B on beach x, fM(i) is the frequency of litter material *i* (HPI, OPI, Foams or NPI) found on beach x, *fTBM* (i, r) is the frequency of taxon B on material i in the region *r* and *Nt (x)* is the total number of rafting biota found on beach *x*.

## Results

### Standardized quantification and categorization of anthropogenic beach litter

**Fig 2:**
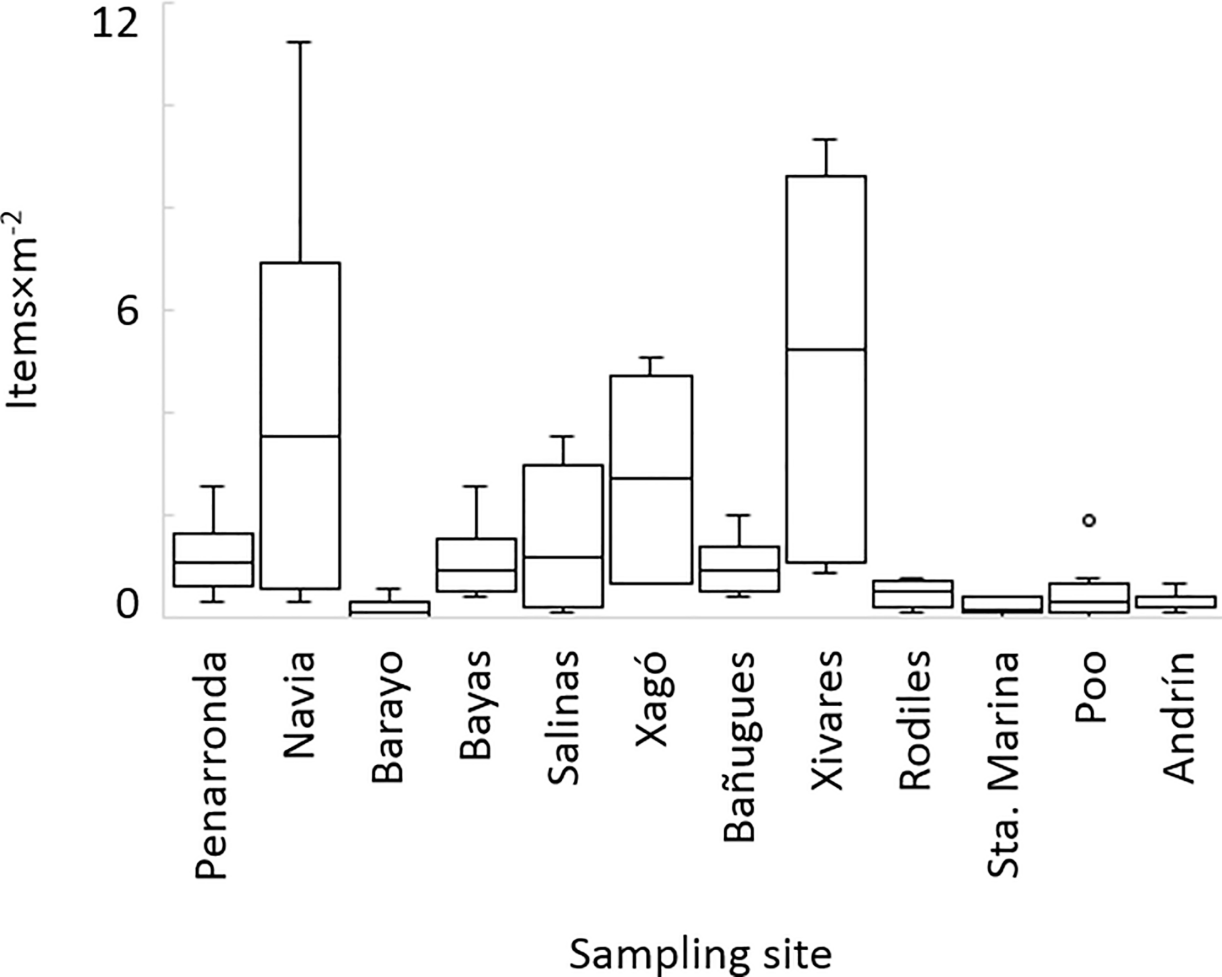
Abundance of anthropogenic litter, counted in a standardized area at the sampled beaches. Data are presented in a box-and whisker plot, with the middle box representing 50% of the values and the upper and lower whiskers representing the values outside of the 50% range. The median and outliers are indicated by a middle line and a circle (◦), respectively. Litter items were counted in a standardized area at each beach.

All the sampled beaches were polluted with anthropogenic litter. The mean abundance of anthropogenic litter ranged from 0.17 ± 0.21 items×m^-2^ (Barayo) to 5 ± 3.95 items×m^-2^ (Xivares). The abundance of anthropogenic litter varied strongly, not only between beaches, but also between quadrats within beaches, indicating a patchy distribution (Fig 2). The composition of beached litter in the region was not significantly different of the composition of litter rafts with biota (Table 4: PERMANOVA 1).

**Table 4:** Detailed results of PERMANOVA analyses.

PERMANOVA	Variable	Factor	df	SS	MS	Pseudo-F	P(perm)	Unique perms
1	Material composition	General litter vs Rafts	1	809.71	809.71	2.6639	0.073	998
		Residuals	22	6687	303.96			
		Total	23	7496.7				
2	Litter abundance,	Beaches	11	382.66	34.788	8.7906	0.001	998
	composition	Residuals	84	332.42	3.9574			
		Total	95	715.08				
3	Litter abundance,	Beach groups	2	224.28	112.14	21.2500	0.001	999
	composition	Residuals	93	490.8	5.2774			
		Total	95	715.08				
4	Litter source	General litter vs Rafts	1	5573.5	5573.5	6.1282	0.003	995
		Residuals	22	20009	909.49			
		Total	23	25582				
5	Attached biota	Raft material	3	30743	10248	2.7185	0.001	998
		Residuals	87	3.2795E5	3769.5			
		Total	90	3.5869E5				

Df = degrees of freedom, SS = sum of squares, MS = mean sum of squares, Pseudo-F = F value by permutation, perm = permutation.

The highest pollution levels were found in direct proximity to the coastal region´s main industrial and populational centers, Gijón (Xivares beach: 5 ± 3.95 items×m^-2^) and Avilés (Salinas and Xagó beaches: 2 ± 1 items×m^-2^ and 2.7 ± 1.9 items×m^-2^, respectively) both of which have a national port and a sewage treatment plant, as well as at the river mouth of the Navia river, in proximity to a fishing port and a marina (Navia beach: 4.3 ± 4 items×m^-2^, see map in Fig 1). The abundance of beach litter at the other sampled beaches along the Cantabrian coastline seems to reflect the geomorphology of the coastline and its exposure to the prevailing eastward surface current, with a maximum peak in the northernmost Cape Peñas: Pollution rose from Barayo eastwards up to Xagó, situated on the western side of Cape Peñas, which is more exposed to the eastward surface current, and subsequently declined on the eastern side of the cape, which is more protected from the prevailing currents (Fig 1, Fig 2).

**Fig 3:**
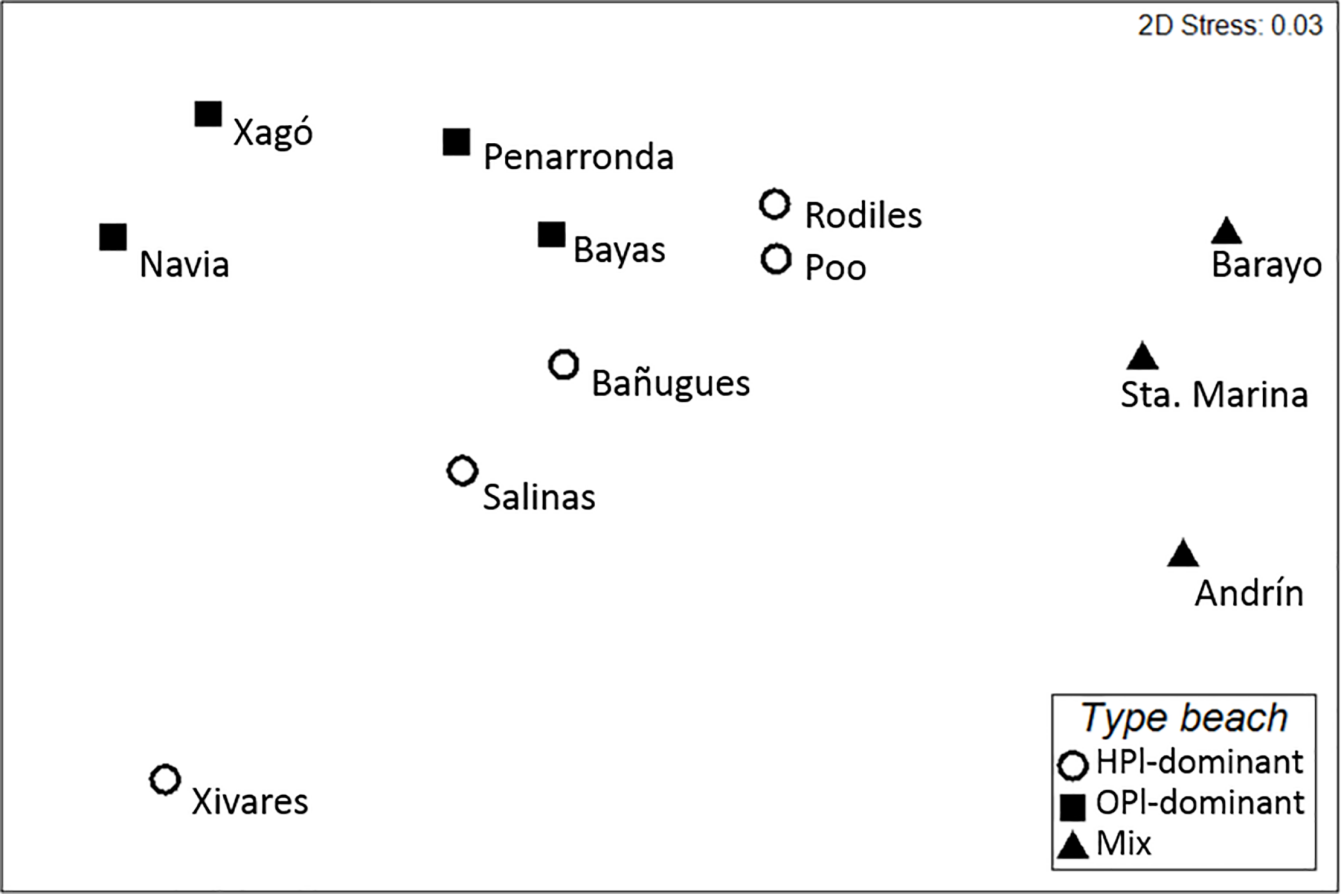
Multi-dimensional plot of the sampled beaches, based on abundance and composition of anthropogenic litter counted in a standardized area at each beach. HPl = hard plastics, OPl = Other plastics, Mix = beaches with mixed litter composition and less than 25 litter items in the standardized sampling area (> 0.35 items×m^2^).

Plastics (including foams) made up the highest share of anthropogenic litter on all beaches (75% to 100%), except at Andrín beach, where non-plastic litter was more abundant (55%; Table 5). The sampled beaches differed significantly from each other regarding both abundance and composition of anthropogenic litter (Table 4: PERMANOVA 2). Beaches were classified based on the prevalent litter material, forming three groups in the sampling area that significantly differed from each other (Table 4: PERMANOVA 3) and could be graphically distinguished by multidimensional scaling (MDS; Fig 3). The treatment of beaches in categories facilitated further analyses.

**Table 5:** Composition and likely source of anthropogenic beach litter from standardized beach litter counts (in white, at left), and fouled litter items along the whole beach area (in grey, at right).

		Anthropogenic beach litter (from standardized sampling in quadrats)										Fouled items (from whole beach area)									
			MATERIAL [%]					SOURCE [%]				MATERIAL [%]					SOURCE [%]			
Beach group	Beach	Litter [items *m^-2^]	HPl	OPl	Foam	NPl	∑Pl	S	F	HH	N/A	Fouled vectors [total]	HPl	OPl	Foam	NPl	∑Pl	S	F	HH	N/A
Mix	Andrín	**0.31**	23	5	18	**55**	45	0	0	0	100	**5**	40	40	20	0	**100**	0	20	**60**	20
Mix	Sta. Marina	**0.22**	44	6	31	19	**81**	6	0	**13**	81	**2**	50	50	0	0	**100**	0	0	**50**	50
Mix	Barayo	**0.17**	58	17	0	25	**75**	0	**8**	**8**	83	**5**	40	20	40	0	**100**	0	**40**	0	60
OPl-dom	Xagó	**2.68**	18	74	8	0	**100**	**34**	**24**	0	41	**4**	75	0	25	0	**100**	0	**75**	0	25
OPl-dom	Penarronda	**1.25**	43	54	2	0	**100**	3	**42**	0	54	**20**	75	10	10	5	**95**	0	**25**	10	65
OPl-dom	Bayas	**1.15**	41	45	10	5	**95**	**8**	4	2	86	**7**	71	14	14	0	**100**	0	**33**	0	67
OPl-dom	Navia	**4.25**	26	68	1	6	**94**	**17**	5	6	72	**1**	100	0	0	0	**100**	0	**100**	0	0
HPl-dom	Salinas	**1.50**	80	11	7	2	**98**	**13**	3	3	81	**25**	48	20	20	12	**88**	0	4	**12**	84
HPl-dom	Xivares	**5.00**	89	3	8	1	**99**	**18**	2	0	80	**4**	75	25	0	0	**100**	0	**25**	**25**	50
HPl-dom	Bañugues	**1.03**	72	14	12	3	**97**	**9**	7	5	78	**2**	100	0	0	0	**100**	0	0	0	100
HPl-dom	Rodiles	**0.47**	71	21	6	3	**97**	**9**	6	3	82	**8**	88	0	13	0	**100**	0	**75**	0	25
HPl-dom	Poo	**0.50**	78	11	8	3	**97**	**19**	3	6	72	**1**	100	0	0	0	**100**	0	0	0	100
X	Silencio	**x**	x	x	x	x	x	x	x	x	x	**4**	100	0	0	0	**100**	0	**25**	0	75
X	Nieva	**x**	x	x	x	x	x	x	x	x	x	**1**	100	0	0	0	**100**	0	0	0	100
X	Figueras	**x**	x	x	x	x	x	x	x	x	x	**5**	0	80	0	20	**80**	0	0	**20**	80
	**MEAN**		54	27	9	10	**90**	**11**	9	4	76		71	17	9	2	**98**	0	**28**	12	60
	**ST. DEV.**		24	26	8	16	16	10	12	4	15		30	24	12	6	6	0	32	19	32

x = no data available. dom = dominant, HPl = Hard plastics, OPl = Other plastics, NPl = Nonplastic, S = Sewage, F = Fishing and aquaculture, HH = Household and leisure, N/A = Not attributable, ST. DEV = Standard deviation.

Most anthropogenic litter items found on the sampled beaches could not be attributed to a source, as many of them were small fragments. For the objects that could be likely assigned to a source, most were sewage-related. At Xagó and Penarronda fishing and aquaculture activities were also important sources of beached litter (Table 5).

### Anthropogenic litter items used as rafts

**Fig 4:**
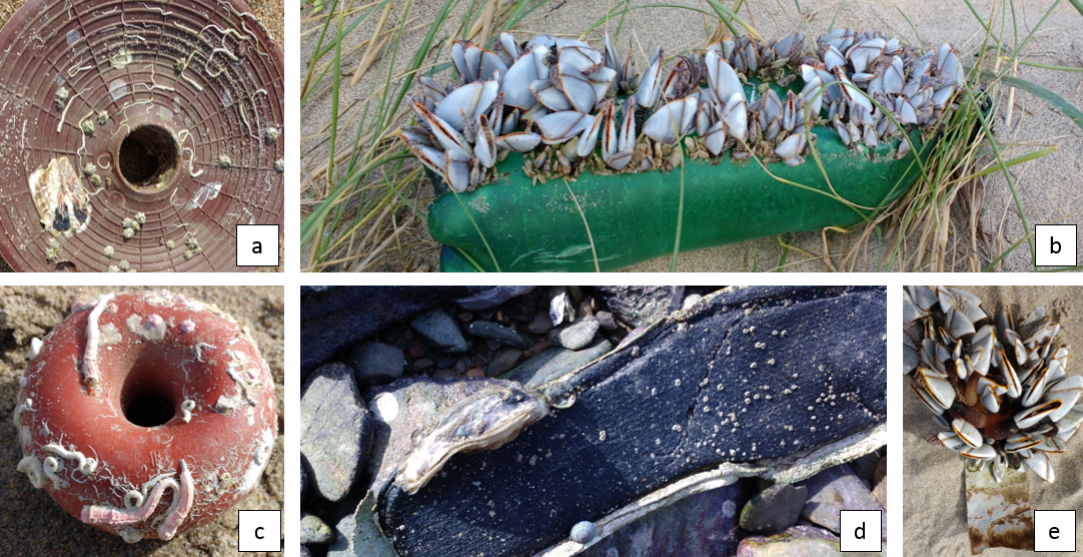
Examples of fouled litter items. a) Hard plastic object with oyster, polychaetes and acorn barnacles b) PET bottle with goose barnacles c) float of fishing net with bryozoan colonies and polychaetes, d) shoe sole with oyster, snail and acorn barnacles, e) duct tape with goose barnacles.

A total of 94 litter objects with attached fauna were found on the surveyed beaches (Fig 4). High prevalence of hard plastics and plastics in general (71 ± 30% and 98 ± 6%, respectively), was found among rafting vectors, while the share of non-plastic objects was very low (2 ± 6%, Table 5). In fact, only five non-plastic objects with attached fauna were found on three beaches: three glass bottles (one with a metal cap), one piece of processed wood, and one sandal, which was counted as nonplastic as the attached organism was found on its textile part. Within the plastics the share of other plastics tended to be less abundant in rafting vectors than in general beach litter (17 ± 24% versus 27 ± 26%), while the share of foams was rather similar in rafting vectors and general litter (9 ± 12% and 9 ± 8%, respectively). The standard deviation between beaches however was high (Table 5).

The main sources of fouled litter items were significantly different from the main sources of other non-fouled beach litter (Table 5, Table 4: PERMANOVA 4). SIMPER showed that the source category with the highest contribution to the differences (after unidentified litter NA, contribution: 37%) was Fishing and Aquaculture (contribution: 34%; Table 6). This particularly important role of fishing/aquaculture related litter for the rafting of biota in the sampling area was especially noticeable at the beaches of Xagó, Navia and Rodiles, where all the identifiable items with attached biota were from this source (Table 5). Leisure and household-related items also had a high share in rafting vectors. Items from this source were found on six beaches and consisted of 20 shoes/sandals and one cosmetic container. Leisure and household was the main litter source for Andrín beach (Table 5). On the other hand, sewage-related litter made up to 11% (mean) of all anthropogenic beach litter, although none of the biota rafts was related to this source (Tables 5 and 6).

**Table 6:** Contributions of several litter sources to the differences between the general beach litter counted in a standardized area (group General) and litter used as biota raft (group Rafts), calculated by SIMPER analysis.

Groups General & Rafts						
Average dissimilarity = 44.61						
	General	Rafts				
Litter source	Abundance	Abundance	Dissimilarity	Diss. / SD	Contribution [%]	Cumulative [%]
Not identified	75.83	53.83	16.67	1.45	37.37	37.37
Fishing/Aquaculture	8.67	33.08	15.36	1.06	34.44	71.81
HH / Leisure	3.83	13.08	6.90	0.77	15.47	87.28
Sewage	11.33	0.00	5.68	1.22	12.72	100.00

HH = household, Diss. = Dissimilarity, SD = standard deviation

### Fauna attached to anthropogenic rafts

**Fig 5:**
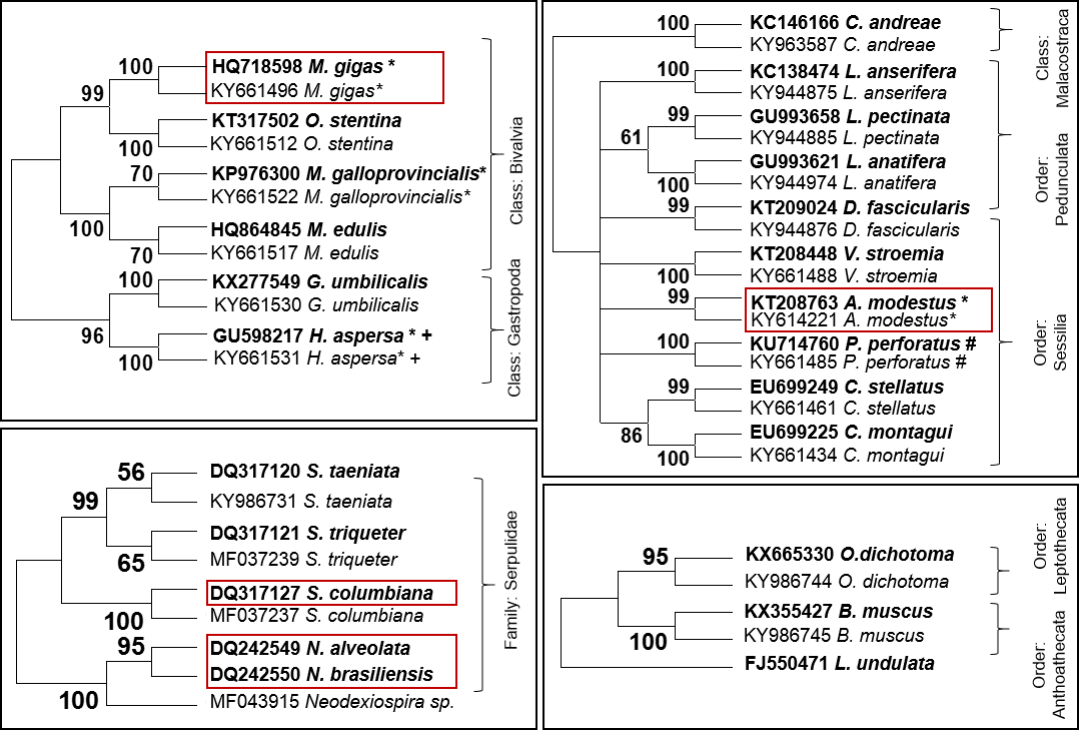
Phylogenetic trees reconstructed from sequences obtained in this study and reference sequences from GenBank database (bold style). a) molluscs, b) crustaceans, c) polychaetes, d) hydrozoans. Frame = Species not native to the study area; * = Species listed in the invasive species database; + = Terrestrial species; # = reference without species voucher.

More than 3300 individuals (or colonies for bryozoans and hydrozoans) were found attached to the litter objects found in the beaches surveyed (Table 7). With genetic analyses, more than 400 DNA barcodes were obtained, identifying 23 species of attached animals from four phyla (Fig 5, Table 7). The Barcodes were submitted to GenBank database, where they are available with the Accession Numbers KY607884-KY607909, KY614195-KY614223, KY628986, KY661434-KY661534, KY683467-KY683511, KY944812-KY944984, KY963587-KY963595, KY986731-KY986745, MF037237-MF037246, MF043915. Crustaceans (Phylum Arthropoda) such as Lepadidae (Goose barnacles), Balanidae and Verrucidae (Barnacles), and the amphipod *Caprella andreae* were the most abundant animals in this study (> 1000 individuals; Table 7), followed by annelids, which all belonged to the family Serpulidae (~700 individuals). Hydrozoan and bryozoan colonies were also very numerous (~400) and might be underestimated in this study, due to the difficulty of counting them individually. As most of the colonies were dried out and in a state of advanced degradation, DNA was degraded in most cases and only two species of Cnidarians were identified from genetic techniques: *Bougainvillia muscus* and *Obelia dichotoma*. The animals found in the present study were morphologically diverse and it is possible that the hydrozoan and bryozoan colony group actually included more species and taxa. Around 100 molluscs were found attached to anthropogenic litter items, with the majority of them belonging to the genus *Mytilus*, followed by the oysters *Crassostrea gigas* and *Ostrea stentina*. Moreover, we found two species of gastropods: the marine species *Gibbula umbilicalis*, and the land snail *Helix aspersa*. For the latter, which is terrestrial, taking into account its common occurrence in the sampled area, it seems likely it did not arrive on the beach by rafting but from the land.

**Table 7:** Overview of species attached to stranded litter, identified in the present study.

*Visual identification*	*N*	*Phylum/ Subphylum*	*Class*	*Order*	*Family*	*Genetic identification*	*Barcodes*	*Geographic origin*
*Goose barnacles*	676	Arthropoda/Crustacea	Maxillopoda	Pedunculata	Lepadidae	*Lepas anatifera*	170	COS
						*Lepas anserifera*	2	
						*Lepas pectinata*	44	
						*Dosima fascicularis*	3	
*Acorn barnacles*	308	Arthropoda/Crustacea	Maxillopoda	Sessilia	Balanidae	Austrominius modestus*	**57**	**Australia, NZ**
						*Chthamalus stellatus*	30	NAT
						*Chthamalus montagui*	26	
						*Balanidae sp*., *(Perforatus perforatus)*	2	
					Verrucidae	*Verruca stroemia*	4	
*Caprellids*	75	Arthropoda/Crustacea	Malacostraca	Amphipoda	Caprellidae	*Caprella andreae*	10	COS
***∑ ARTHROPODS***	**1059**							
*Mytilidae*	70	Mollusca	Bivalvia	Mytiloida	Mytilidae	*Mytilus edulis*	5	NAT
						Mytilus galloprovincialis*	1	
						*Mytilus sp*.	10	x
*Ostreidae*	21	Mollusca	Bivalvia	Ostreoida	Ostreidae	Crassostrea gigas*	**16**	**NE-Pacific**
						***Ostrea stentina***	**1**	**S-Atlantic, Med**
*Gastropods*	2	Mollusca	Gastropoda	x	Trochidae	*Gibbula umbilicalis*	2	NAT
***∑ MARINE MOLLUSCS***	**93**							
*Polychaetes*	699	Annelida	Polychaeta	Canalipalpata	Serpulidae	*Spirobranchus triqueter*	3	NAT
						*Spirobranchus taeniatus*	17	
						***Serpula columbiana***	**1**	**N-Pacific**
						***Neodexiospira sp*.**	**1**	**S-Atlantic**
						*Spirobranchus sp*.	3	x
***∑ ANNELIDS***	**699**							
*Hydrozoan and Bryozoan colonies*	396	Cnidaria	Hydrozoa	Anthoathecata	Bougainvilliidae	*Bougainvillia muscus*	1	NAT
				Leptomedusae	Campanulariidae	*Obelia dichotoma*	1	COS
***∑ HYDROZOANS + BRYOZOANS***	**396**							
*Gastropod, terrestrial*	4	Mollusca	Gastropoda	x	Helicidae	Helix aspersa aspersa*	4	NAT

N = total number of individuals found, NAT = native, COS = cosmopolitan distribution, N = North, S = South, Med = Mediterranean sea. Non-native species are marked by bold writing.

* = Species (both native and non-native to study area) listed in the global invasive species database (GISD, http://www.issg.org/database).

Most of the rafting animals were native to the study region or recognized as cosmopolitans (Lepadidae). Five species were not native: *Crassostrea gigas*, *Ostrea stentina*, *Austrominius modestus*, *Serpula columbiana*, and *Neodexiospira sp*. *C*. *gigas* and *A*. *modes*tus are listed in the global invasive species database (GISD, http://www.issg.org/database). The native *M*. *galloprovincialis* and the terrestrial species *H*. *aspersa* are included in GISD as well. The species identification provided by BLAST was confirmed from phylogenetic analysis after clustering analyses including voucher species references from GenBank (Fig 5).

**Fig 6:**
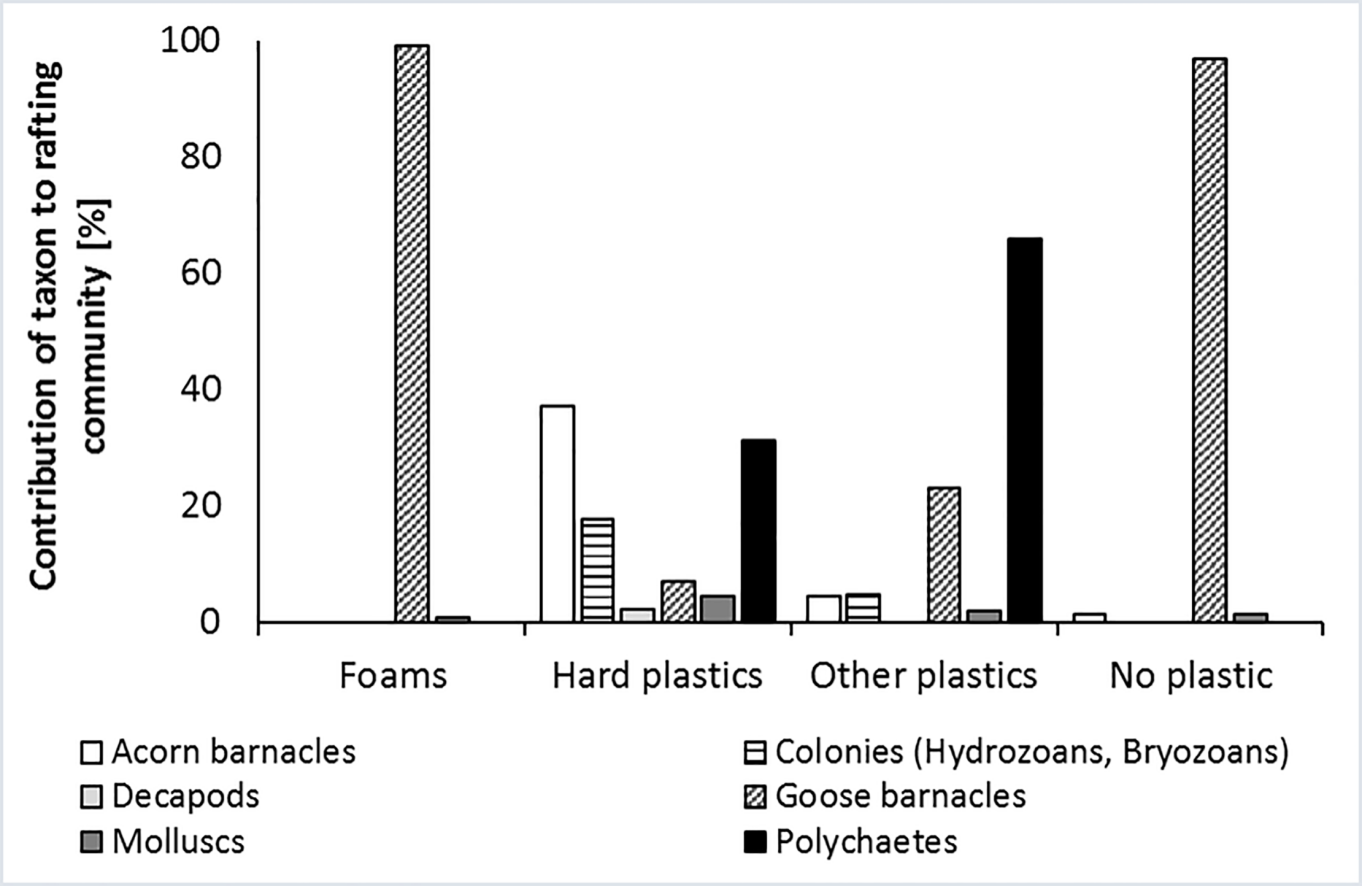
Particular profile of attached biota for each litter material.

Regarding the type of material carrying each species, differences occurred in this region between taxonomic groups. While molluscs like *Mytilus* and *Crassostrea* were found on all types of anthropogenic litter, Polychaetes were exclusively found on hard plastic and other plastic items. Barnacles, like *Austrominius*, were found on all materials except foams, but were most important on hard plastic items. Therefore, each type of litter seemed to exhibit a particular profile of attached biota (Fig 6). Foams carried almost exclusively goose barnacles (99%) and, to a much lesser extent, molluscs (1%). Non-plastic items contained a similar biota profile, with an additional small share of barnacles (2%). Hard plastic and other plastic objects on the other hand carried a broad spectrum of attached taxa. On hard plastic items the main share of attached biota were barnacles (37%), polychaetes (31%) and bryozoan colonies (18%). They also carried goose barnacles, molluscs, and decapods (7%, 4%, and 2%, respectively). On other plastics, the main share of attached biota was made up of polychaetes (66%) and goose barnacles (23%), while barnacles, bryozoan colonies, and molluscs were less common (5%, 5%, and 2%, respectively). Differences between materials regarding the biota profile were indeed highly statistically significant (Table 4: PERMANOVA 5).

### Inference of litter-related biotic community from beach litter composition

**Fig 7:**
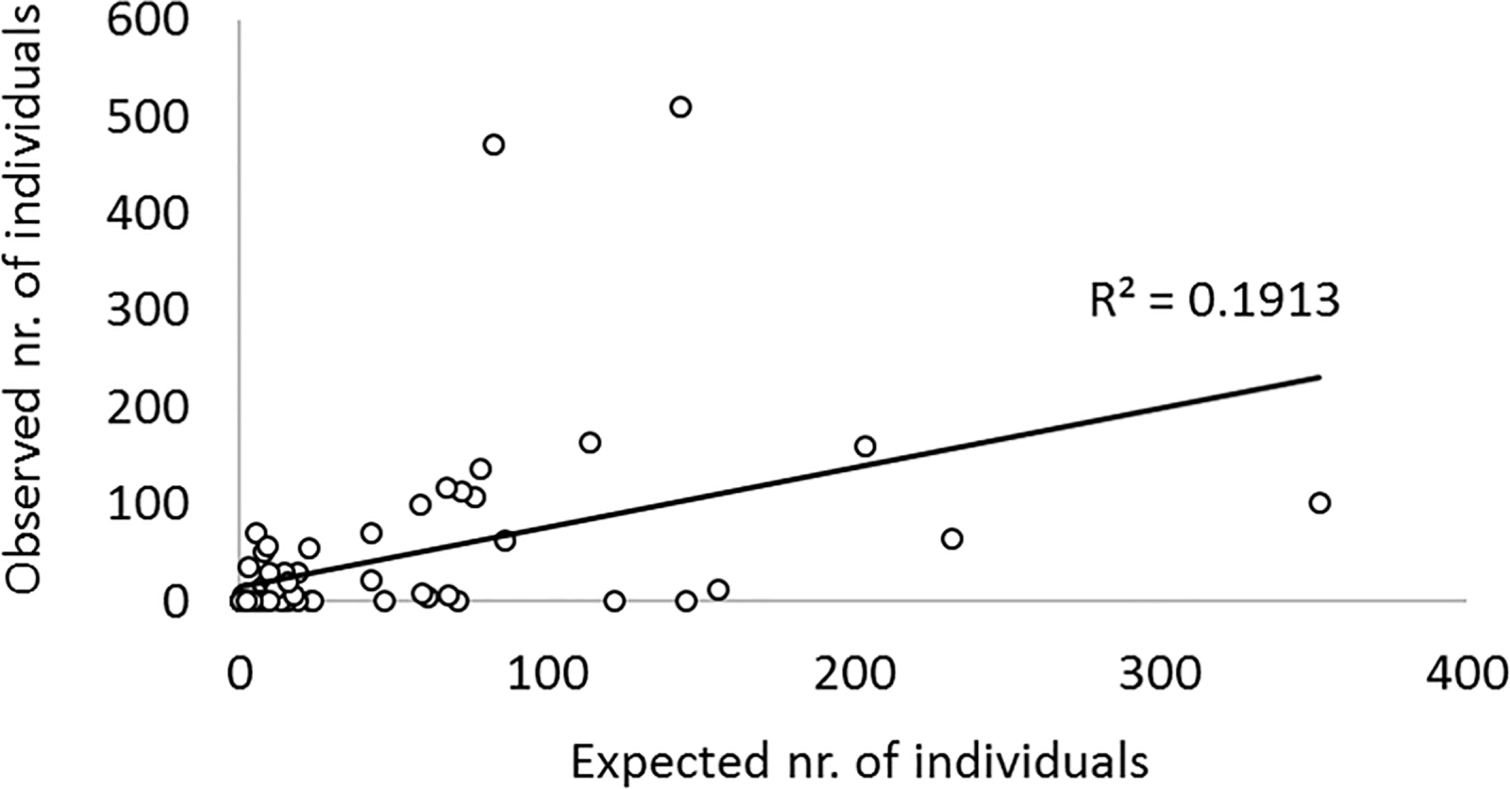
Correlation between expected and observed numbers of individuals of several taxa, calculated from data of 12 Asturian beaches.

We tested if the composition of an area´s macrobiotic communities attached to stranded litter items can be predicted based on its composition of anthropogenic beach litter, using the data of the 12 beaches where standardized litter counts have been conducted. The predicted frequency of attached biota of several taxa, estimated from litter composition significantly correlated with the actually observed frequencies on both sides of cape Peñas (Western side: Spearman`s rank correlation coefficient (R) = 0.498; *p* = 0.002; Eastern side: R = 0.629; *p* = 0.027), as well as for the whole sampling area (R = 0.565; *p* < 0.001; Fig 7). For the exact figures of estimated and observed biota, please see the Supporting table (S1 Table).

## Discussion

In this study six rafting species were recorded for the first time on anthropogenic beach litter: *Verruca stroemia*, *Ostrea stentina*, *Gibbula umbilicalis*, *Spirobranchus taeniata*, *Serpula columbiana*, and *Neodexiospira sp*. Although many rafting species have been documented on anthropogenic marine litter during the last years [16] and the recent discovery of 289 living marine species, which had crossed the Pacific Ocean on objects detached by a tsunami, showed the importance of floating marine litter as a rafting vector [71], many rafting species are not known or reported yet and knowledge of the actual dimension and impact of marine litter rafting is still far from complete. The finding of *Perforatus perforatus* on anthropogenic litter is particularly interesting, as large numbers of this species, probably originating from NW Spain, have been found on beach litter in Wales [72]. A similar range expansion might also occur for invasive barnacles, such as *Austrominius modestus*.

Besides the species listed above, most of the taxa found in our study are known rafters and have already been found on anthropogenic litter (floating or stranded) in other regions [16]. The predominance of cosmopolitan stalked barnacles among marine rafters is a common phenomenon, with the small and light-weight species *L*. *pectinata* and *D*. *fascicularis* being especially suited for the colonization of smaller rafts [16,23]. *Lepas* barnacles may influence the rafting community on plastic debris: the ratio *Lepas* cover /surface area was found positively correlated with the diversity of mobile rafters, while negatively with sessile rafters’ diversity, in a study by Gil and Pfaller (2016). Our results were concordant with this study, since the debris dominated by goose barnacles contained a very low diversity of other sessile rafting species (only molluscs and acorn barnacles), while materials with a lower share of goose barnacles exhibited a relatively diverse attached community (Fig 6). Another common rafter found in this study was the amphipod *Caprella andreae*. The genus *Caprella* is generally adapted to rafting because of their reduced abdominal appendages, and *C*. *andreae* is the only known obligate rafter in its genus [60].

Two non-native oysters were found on Figueras beach, close to the region´s only active site of mollusc aquaculture. While *C*. *gigas* is a recognized invasive species and quite common along the Asturian coast, *O*. *stentina* has only been reported in the region once before, in the port of Avilés [49]. These two findings with a linear distance of less than 100 km may indicate that this species is already established in the region, and may use anthropogenic litter for dispersal beyond the range of its propagules. The results show a link between the composition of anthropogenic beach litter in an area and the frequency of several taxa of fauna attached to stranded litter objects. This finding should be valid for a broad range of coastal regions, as it is based on taxa composition and general litter materials, rather than on particular species and/or litter items, which may vary more strongly between regions.

The strong prevalence of (hard) plastic rafts confirms the results of previous studies [73]. The very low share of non-plastic rafts may be due to the fact that the majority of these items are not buoyant and/or of very little persistence. Plastic foams, despite being highly buoyant and having rather rough surfaces, which facilitate initial colonization [16], are less stable and persistent than hard plastics [29]. This may explain their low share amongst rafting vectors. For the potential sources of litter with rafting biota, there was a high share of unidentified items but still some important conclusions may be drawn from our results. Firstly, rafting vectors could be identified and attributed to a source much more frequently than other items of anthropogenic beach litter. The reason is probably that small plastic fragments whose source cannot be identified, which are quite common in beach litter in general, are too small for serving as rafts. Fazey and Ryan (2016) proposed size and buoyancy as predictors of dispersal distance for floating debris [74]. Given that biofouling reduces an item´s buoyancy, smaller items will sink faster than bigger items and travel much smaller distances [75]. This phenomenon may also explain why sewage litter, although quite abundant on beaches, was never found as a rafting vector. Rafting vectors from fishing and aquaculture, as well as other sea-based activities, have been reported in other studies [41,76]. An explanation for the high occurrence of items from these sources among rafts may be their buoyancy, stability, size and persistence. 12 of the 23 fishing/aquaculture-related rafting vectors were buoys or netfloats, which are obviously highly buoyant and seven were grids or cages made from stable plastic wire, which are big items with a rather small surface/volume ratio. The other four rafts were rather big items (min. 10x2x2 cm^3^) made from hard plastics. Leisure and household-related litter is quite difficult to define, because many of the items which might stem from this source might as well stem from sea-based sources (e.g. PET bottles). These items have not been assigned to a source category, so perhaps the actual contribution of this source was higher. Shoes and sandals, clearly sourced household or leisure, are known to be able to float over large distances and have already been reported as rafting vectors [44,77–79].

The patchy abundance of beach litter, with high variances both within and between beaches was congruent with the situation reported in many other studies [7,9,80]. Although comparisons of abundance between different locations, observers, and studies with different approaches (regarding for example transect size, choice of strand lines and/or ground between strand lines sampled, minimum size of items counted, biological material present in the sampled area etc.) are rather difficult [7,65,81], the abundance of beach litter found in this study falls within the same range as reported for many other sampling sites around the globe. As this study focuses on stranded litter which had already been at sea, the litter counts were conducted in transects targeting tidelines, where natural and anthropogenic litter is deposited by the sea. Targeting areas of litter accumulations, the results are likely overestimating the total litter abundances of the sampled beaches, and are not representative for the whole area of the beaches. They do however allow for comparisons of stranded litter abundances between the beaches sampled during this study, where the same method was used for all beaches.

Plastics (including plastic foams) are reported as the main constituents of beach litter in most studies [7]. According with that, the share of plastics found on beaches along the Cantabrian coast (present study) was rather high. Source attribution of the stranded litter items was a difficult task because the majority of items could not be clearly related to a litter category, either because the item could stem from several sources, or because the item was not identifiable (i.e. fragments). Notwithstanding it, our results indicate that sewage-related litter is a problem in the sampled area. In fact, waste-water discharging pipelines and accumulations of preproduction pellets in the sand below such pipelines were noted on several of the sampled beaches (personal observation SR), but did not enter in the present study due to their small size. Fishing and aquaculture have also been identified as important litter sources in the sampling area. This finding is consistent with the fact that pollution by lost or discarded fishing gear is a common problem in the world’s seas (including the benthos) and on beaches [37,82–84]. There is a high activity of small-scale fishery, with 19 fishing ports along Asturias coastline and a large area of fishing grounds near- and off-shore, plus one active site of mollusc aquaculture (mainly oysters) near Figueras, and several crustacean ponds (http://www.sigmarinoasturias.es/).

The exposure to the prevalent currents may make the sampling area a sink for anthropogenic floating litter and attached biota from other areas. In fall and winter, the sampling area is dominated by a warm poleward surface current, referred to as ‘Navidad’, which enters near Cape Finisterre and moves eastward along the Cantabrian shelf and slope [51]. As the samplings presented in this study were conducted from mid-February to mid-March, it could be assumed that the overall accumulation pattern, particularly the increase of litter abundances from more western beaches towards the tip of Cape Peñas, was driven by this current. On the eastern side of cape Peñas, sediments are transported from the coastal currents to the beaches [85]. This transport may explain the observed abundances of litter on these beaches, which are not directly exposed to the prevalent current. Apart from this main driver, there seems to be an effect of rivers in the area, contributing to the high litter abundance on the beaches Navia and Xivares. Both are situated at the mouth of rivers (Rio Navia and Rio Aboño, respectively). Riverine influence was also reflected in the relatively high share of sewage-linked litter on both beaches.

Although the present study clearly showed the relation between anthropogenic beach litter composition and attached fouling biota in a coastal area, it had some limitations. The samplings were restricted to one geographic area (the south-central Bay of Biscay) and season (february to march), and each beach was sampled only once. Moreover, our study concentrated on stranded anthropogenic litter and did not include litter which was still floating in the water. Thereby we ensured to sample only taxa/species which are still present after a beaching event and might therefore pose a risk of invasion. On the other hand, it should be considered that the biota found on beach litter in this study probably do not represent the complete macrobiotic rafting community of the respective items before the beaching event, as beached litter is often biased towards sessile biota [16].

In summary, the results presented here give several important insights in the mechanisms on biota rafting on anthropogenic marine litter. Plastic items, except for foams, house a much more diverse biota community than non-plastic items and foams, which may be due to their stability and buoyancy. Several non-native and invasive species were present on litter items along the sampled beaches. Aquaculture and fishing activities were a major source of biota rafts, while sewage discharge was the most important source of all anthropogenic beach litter in the study region. We found that the frequency of a specific taxon of rafting biota in a coastal area may be predicted based on each litter material’s characteristic biota profile and the beaches’ litter composition. This approach, after refined and tested from more regions, could serve as a simple and cost-efficient tool for risk assessment in the future.

## Supporting information

S1 Fig Sampling scheme. (TIF)

S1 Table Expected vs. observed numbers of each taxon of the community attached to stranded litter. (DOCX)
