# Supplementary figures and images for: Anthropogenic marine litter composition in coastal areas may be a predictor of potentially invasive rafting fauna
Source: PLoS One. 2018 Jan 31;13(1):e0191859. doi: 10.1371/journal.pone.0191859 (PMC5792010; doi:10.1371/journal.pone.0191859)

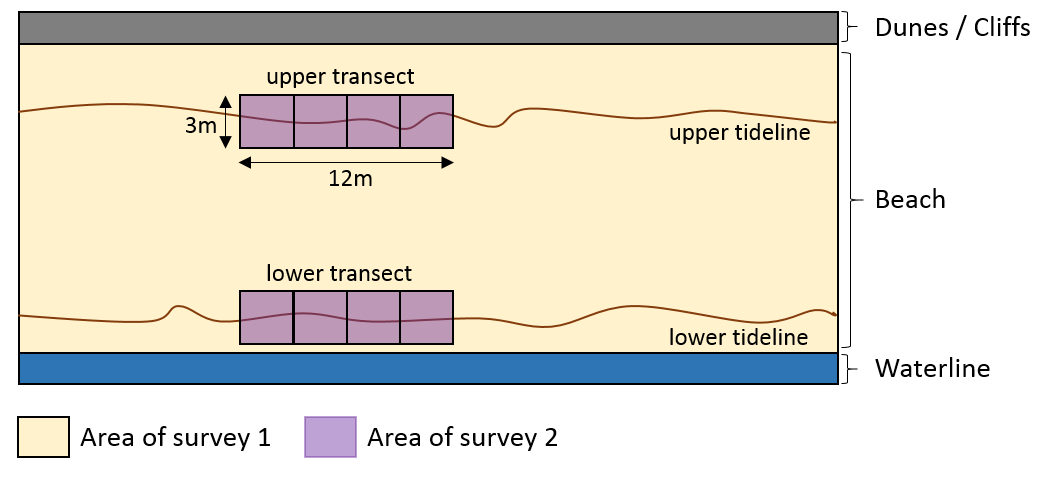

Supplement: S1 Fig — (TIF) [file pone.0191859.s001.tif]
