# Supplementary material for: Anthropogenic marine litter composition in coastal areas may be a predictor of potentially invasive rafting fauna
Source: PLoS One. 2018 Jan 31;13(1):e0191859. doi: 10.1371/journal.pone.0191859 (PMC5792010; doi:10.1371/journal.pone.0191859)
Supplement: S1 Table — (DOCX) [file pone.0191859.s002.docx]

| Beach | Frequency of litter type | | | | ∑Rafting biota on beach | Taxon | Abs. values | |
| --- | --- | --- | --- | --- | --- | --- | --- | --- |
|  | HPl | OPl | Foam | NPl |  |  | exp | obs |
| Penarronda | 0.43 | 0.54 | 0.02 | 0.00 | 416 | Barnacles | 76 | 107 |
|  |  |  |  |  |  | Bryozoa | 43 | 21 |
|  |  |  |  |  |  | Decapods | 4 | 0 |
|  |  |  |  |  |  | GB | 73 | 112 |
|  |  |  |  |  |  | Molluscs | 13 | 17 |
|  |  |  |  |  |  | Polychaetes | 204 | 159 |
| Navia | 0.26 | 0.68 | 0.01 | 0.06 | 36 | Barnacles | 5 | 5 |
|  |  |  |  |  |  | Bryozoa | 3 | 0 |
|  |  |  |  |  |  | Decapods | 0 | 0 |
|  |  |  |  |  |  | GB | 9 | 0 |
|  |  |  |  |  |  | Molluscs | 1 | 1 |
|  |  |  |  |  |  | Polychaetes | 19 | 30 |
| Barayo | 0.58 | 0.17 | 0.00 | 0.25 | 209 | Barnacles | 47 | 0 |
|  |  |  |  |  |  | Bryozoa | 23 | 0 |
|  |  |  |  |  |  | Decapods | 3 | 35 |
|  |  |  |  |  |  | GB | 67 | 116 |
|  |  |  |  |  |  | Molluscs | 7 | 4 |
|  |  |  |  |  |  | Polychaetes | 61 | 4 |
| Bayas | 0.41 | 0.45 | 0.10 | 0.05 | 829 | Barnacles | 143 | 510 |
|  |  |  |  |  |  | Bryozoa | 78 | 137 |
|  |  |  |  |  |  | Decapods | 8 | 15 |
|  |  |  |  |  |  | GB | 232 | 65 |
|  |  |  |  |  |  | Molluscs | 24 | 0 |
|  |  |  |  |  |  | Polychaetes | 352 | 102 |
| Salinas | 0.80 | 0.11 | 0.07 | 0.02 | 482 | Barnacles | 145 | 0 |
|  |  |  |  |  |  | Bryozoa | 71 | 0 |
|  |  |  |  |  |  | Decapods | 9 | 0 |
|  |  |  |  |  |  | GB | 82 | 471 |
|  |  |  |  |  |  | Molluscs | 19 | 0 |
|  |  |  |  |  |  | Polychaetes | 156 | 11 |
| Xagó | 0.18 | 0.74 | 0.08 | 0.00 | 225 | Barnacles | 23 | 54 |
|  |  |  |  |  |  | Bryozoa | 15 | 0 |
|  |  |  |  |  |  | Decapods | 1 | 0 |
|  |  |  |  |  |  | GB | 59 | 100 |
|  |  |  |  |  |  | Molluscs | 5 | 70 |
|  |  |  |  |  |  | Polychaetes | 122 | 1 |
| Banugues | 0.72 | 0.14 | 0.12 | 0.03 | 56 | Barnacles | 15 | 0 |
|  |  |  |  |  |  | Bryozoa | 8 | 51 |
|  |  |  |  |  |  | Decapods | 1 | 0 |
|  |  |  |  |  |  | GB | 13 | 0 |
|  |  |  |  |  |  | Molluscs | 2 | 0 |
|  |  |  |  |  |  | Polychaetes | 18 | 5 |
| Xivares | 0.89 | 0.03 | 0.08 | 0.01 | 16 | Barnacles | 5 | 10 |
|  |  |  |  |  |  | Bryozoa | 3 | 2 |
|  |  |  |  |  |  | Decapods | 0 | 0 |
|  |  |  |  |  |  | GB | 3 | 3 |
|  |  |  |  |  |  | Molluscs | 1 | 0 |
|  |  |  |  |  |  | Polychaetes | 5 | 1 |
| Rodiles | 0.71 | 0.21 | 0.06 | 0.03 | 316 | Barnacles | 86 | 63 |
|  |  |  |  |  |  | Bryozoa | 43 | 70 |
|  |  |  |  |  |  | Decapods | 5 | 0 |
|  |  |  |  |  |  | GB | 59 | 7 |
|  |  |  |  |  |  | Molluscs | 12 | 12 |
|  |  |  |  |  |  | Polychaetes | 114 | 164 |
| Sta. Marina | 0.44 | 0.06 | 0.31 | 0.19 | 14 | Barnacles | 2 | 1 |
|  |  |  |  |  |  | Bryozoa | 1 | 5 |
|  |  |  |  |  |  | Decapods | 0 | 0 |
|  |  |  |  |  |  | GB | 8 | 0 |
|  |  |  |  |  |  | Molluscs | 0 | 0 |
|  |  |  |  |  |  | Polychaetes | 2 | 8 |
| Poo | 0.78 | 0.11 | 0.08 | 0.03 | 50 | Barnacles | 15 | 30 |
|  |  |  |  |  |  | Bryozoa | 7 | 0 |
|  |  |  |  |  |  | Decapods | 1 | 0 |
|  |  |  |  |  |  | GB | 9 | 0 |
|  |  |  |  |  |  | Molluscs | 2 | 0 |
|  |  |  |  |  |  | Polychaetes | 16 | 20 |
| Andrín | 0.23 | 0.05 | 0.18 | 0.55 | 92 | Barnacles | 9 | 57 |
|  |  |  |  |  |  | Bryozoa | 4 | 0 |
|  |  |  |  |  |  | Decapods | 0 | 0 |
|  |  |  |  |  |  | GB | 68 | 5 |
|  |  |  |  |  |  | Molluscs | 2 | 0 |
|  |  |  |  |  |  | Polychaetes | 10 | 30 |
|  |  |  |  |  |  |  |  |  |
